# How eccentricity modulates attention capture by direct face/gaze and sudden onset motion

**DOI:** 10.3758/s13414-025-03015-8

**Published:** 2025-02-06

**Authors:** Jens Kürten, Christina Breil, Roxana Pittig, Lynn Huestegge, Anne Böckler

**Affiliations:** 1 Department of Psychology, University of Würzburg, Röntgenring 11, 97070 Würzburg, Germany; 2 Institut für Gesundheitsförderung und Prävention, Linke Wienzeile 48-52, 1060 Vienna, Austria; 3 Georg-Elias-Müller-Institut für Psychologie, Universität Göttingen, Goßlerstraße 14, 37073 Göttingen, Germany

**Keywords:** Direct face, Direct gaze, Motion, Social attention, Visual attention, Spatial attention, Stimulus eccentricity

## Abstract

We investigated how processing benefits for direct face/gaze and sudden onset motion depend on stimulus presentation location, specifically eccentricity from fixation. Participants responded to targets that were presented on one of four stimuli that displayed a direct or averted face and gaze either statically or suddenly. Between participants, stimuli were presented at different eccentricities relative to central fixation, spanning 3.3°, 4.3°, 5.5° or 6.5° of the visual field. Replicating previous studies, we found processing advantages for direct (vs. averted) face/gaze and motion onset (vs. static stimuli). Critically, while the motion-onset advantage increased with increasing distance to the center, the face/gaze direction advantage was not significantly modulated by target eccentricity. Results from a control experiment with eye tracking indicate that face/gaze direction could be accurately discriminated even at the largest eccentricity. These findings demonstrate a distinction between the processing of basic facial and gaze signals and exogenous motion cues, which may be based on functional differences between central and peripheral retinal regions. Moreover, the results highlight the importance of taking specific stimulus properties into account when studying perception and attention in the periphery.

**Supplementary information:**

The online version contains supplementary material available at 10.3758/s13414-025-03015-8.

## Introduction

Social interactions play a central role in our everyday lives. In order to interpret and react to the utterances and actions of the people around us, we often need to accurately perceive the direction of their social attention, usually displayed by their combined head and gaze orientation. In fact, humans seem to master this skill effectively (Emery, 2000; Tomasello & Carpenter, 2007). We reflexively shift our attention in response to averted gaze cues and spontaneously follow the gaze and/or head orientation of others to external locations (i.e., gaze cueing), providing the basis for joint attention (Driver et al., 1999; Friesen & Kingstone, 1998; Frischen et al., 2007; Stephenson et al., 2021). In real life, social signals like gaze and face direction seldom occur in isolation. Instead, they often paired with other cues that affect our attention and perception. For example, when another person raises their head to look at us, direct gaze co-occurs with sudden onset motion, another strong cue for attention capture (Abrams & Christ, 2005). Previous studies showed that face/gaze direction and sudden onset motion exert their influence independently from each other (Böckler et al., 2014, 2015). In the underlying experiment, participants were required to detect a target that was randomly presented on one of four faces that gazed either directly at the observer or into the periphery (*face/gaze*: direct or averted) and had either made a head movement or not (*motion*: sudden or static). Results showed that target classification was facilitated by both direct face/gaze and motion, but it was most efficient when the two factors coincided in time and space, that is, when the target was presented at the location of a suddenly established direct gaze. Crucially, the effects of direct gaze and motion were additive and were differentially affected by inhibition of return manipulations, indicating independent underlying attentional channels. This notion was substantiated by further studies with the same paradigm (Böckler et al., 2015; Breil, Huestegge et al., 2022a).

In these experiments, the location of the stimuli was held constant, and participants were instructed to fixate on a central cross, thereby ensuring that all stimuli would appear in the close periphery but within foveal boundaries of the visual field (4.3° visual angle [VA]). This aspect is particularly important because the properties of visual processing shift greatly from center to periphery: perception is most accurate and contrast-sensitive in the foveal region while the periphery is known to be particularly sensitive to motion cues (Burnat, 2015; Kitterle, 1986; Yu et al., 2010). However, similar to many other attention phenomena, faces seem to be an exception to the rule: They are processed fairly accurately and often better than other objects even when presented at greater eccentricities (Boucart et al., 2016; Hershler et al., 2010), an advantage that seems especially pronounced for the encoding of emotional expressions (Bayle et al., 2011; Calvo et al., 2014; Rigoulot et al., 2012).

Similar to faces, it has been hypothesized that efficiently processing gaze cues was particularly important for our survival as social beings (Emery, 2000; Frischen et al., 2007). In line with this idea, it seems relevant that we perceive and react not only to people that are directly in front of us but also to those in our immediate surroundings. Previous studies reported that judgements of gaze direction are reliable up to 4 or 5° VA (Loomis et al., 2008; Palanica & Itier, 2017) and that attention orienting in response to gaze cues is accurate when the gaze stimulus is presented at 5°, but no longer at 7.5° (Yokoyama & Takeda, 2019). Hence, while both face recognition and gaze discrimination eventually break down in the far periphery, they seem relatively robust to increasing presentation eccentricity in comparison to other objects. The dependence of processing accuracy on eccentricity may in part rely on stimulus features such as contrast and size, however, size-scaling alone does lead to equal performance with peripheral (compared to foveal) presentation of faces (Jebara et al., 2009; Mäkelä et al., 2001; see Strasburger et al., 2011 for a review). Instead, the relative superiority in peripheral vision observed for faces and gazes seems to depend on their social and semantic category, and varies with task requirements (Huestegge & Böckler, 2016; Jebara et al., 2009; Pitcher & Ungerleider, 2021).

While a direct-face/gaze advantage for centrally and slightly peripherally presented faces has been repeatedly demonstrated (Böckler et al., 2014; Senju & Johnson, 2009), some setups seem to induce averted-face/gaze advantages. For instance, Breil, Huestegge et al. (2022a), Breil, Raettig et al. (2022b) showed faces with an approach-oriented emotional expression (joy) to capture attention when they established direct gaze, while faces with avoidance-oriented expressions (disgust) captured attention when they averted gaze. In addition, Riechelmann et al. (2021) found that, for very brief and central presentations, averted gaze can be detected more easily than direct gaze. Besides task and context specificities, a factor that may be critical is central vs. peripheral processing. Does others’ social attention shape attention and perception differentially at different eccentricities relative to current fixation? Previous findings suggest that attention capture by direct face/gaze may only hold within foveal boundaries (Loomis et al., 2008; Palanica & Itier, 2014, 2015, 2017; Yokoyama & Takeda, 2019), and due to the decrease in resolution ability in the periphery one could expect a breakdown of the direction effect at some point. However, this remains to be tested empirically. The first aim of the present study is therefore to determine how attention capture by a direct (vs. averted) face and gaze is affected by presentation eccentricity and to specify the boundary conditions of such a modulation.

In contrast to object or face/gaze processing, the detection of motion seems to be preserved even at high eccentricities, most likely due to greater retinal rod density, which facilitates foveal attention shifts in order to initiate appropriate reactions to potential threats in the environment (Burnat, 2015; Kowler, 2011). Based on this notion, we hypothesize that sudden onset motion of faces should exert an equally strong or even stronger influence on attention allocation when stimuli are presented more peripherally than in the study by Böckler et al. (2014). However, it is less clear to what extent sudden onset face motion captures attention at smaller (vs. larger) eccentricities. The second aim of the present study is to systematically investigate the modulation of the motion onset advantage by presentation eccentricity.

**Fig. 1:**
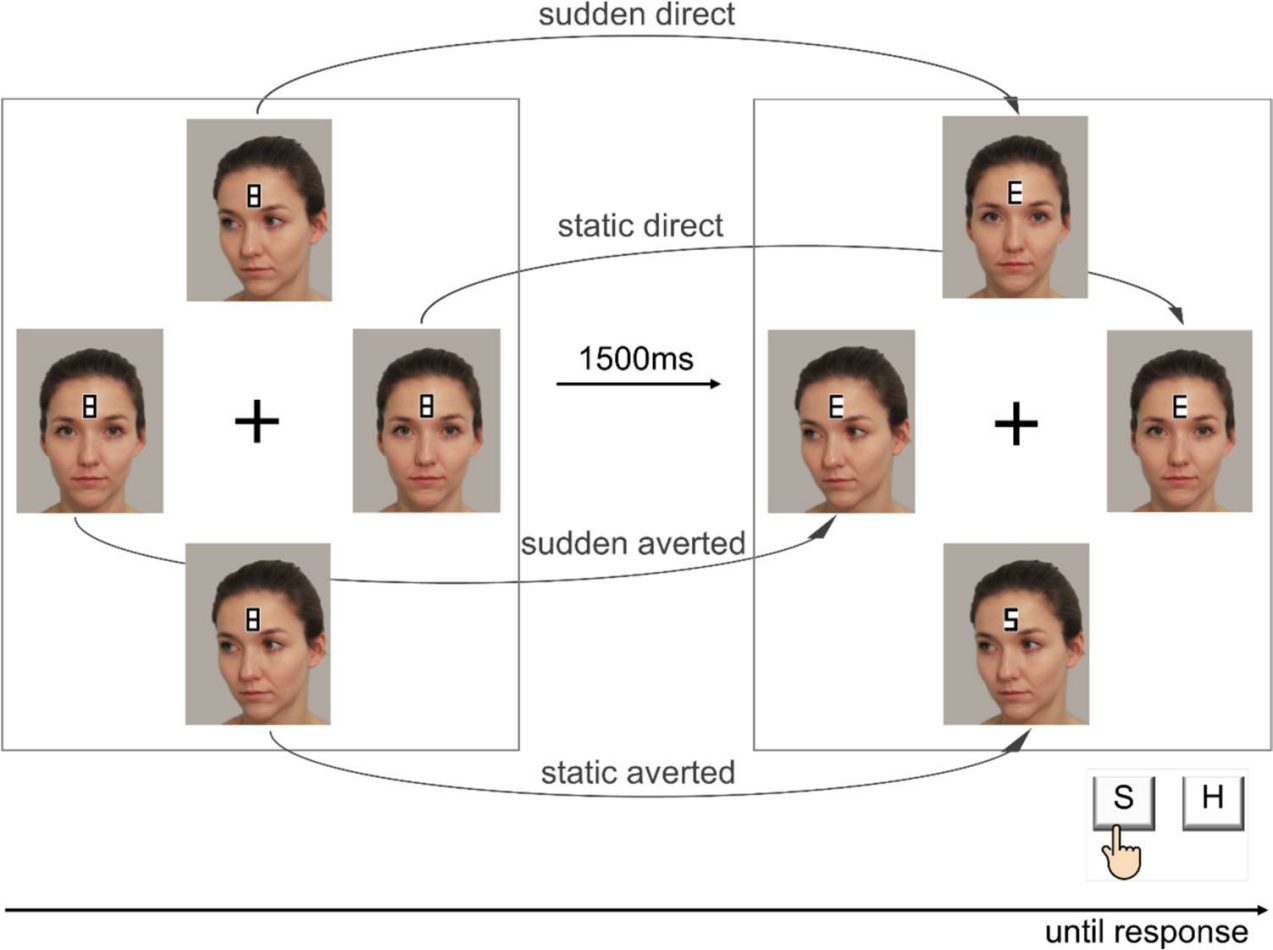
Sample trial sequence of group D2 (eccentricity = 4.3°). *Note.* At the beginning of each trial, number-8-figures were presented at the forehead of all four faces. After 1500 ms, they were replaced by one target and three distractor letters while, at the same time, one direct stimulus changed to averted and one averted stimulus changed to direct. The other two stimuli remained unchanged, resulting in the four experimental conditions sudden direct, static direct, sudden averted, static averted. Participants were required to react to the target letter by pressing the corresponding button on the keyboard as fast and accurate as possible. The original eccentricity of 4.3°VA from Böckler et al. (2014) was adopted for group D2 as visualized in Fig. 1 and was varied for the remaining groups as shown in Fig. 2. All parts of this figure are original. Photographs were taken by the authors and printed with permission

**Fig. 2:**
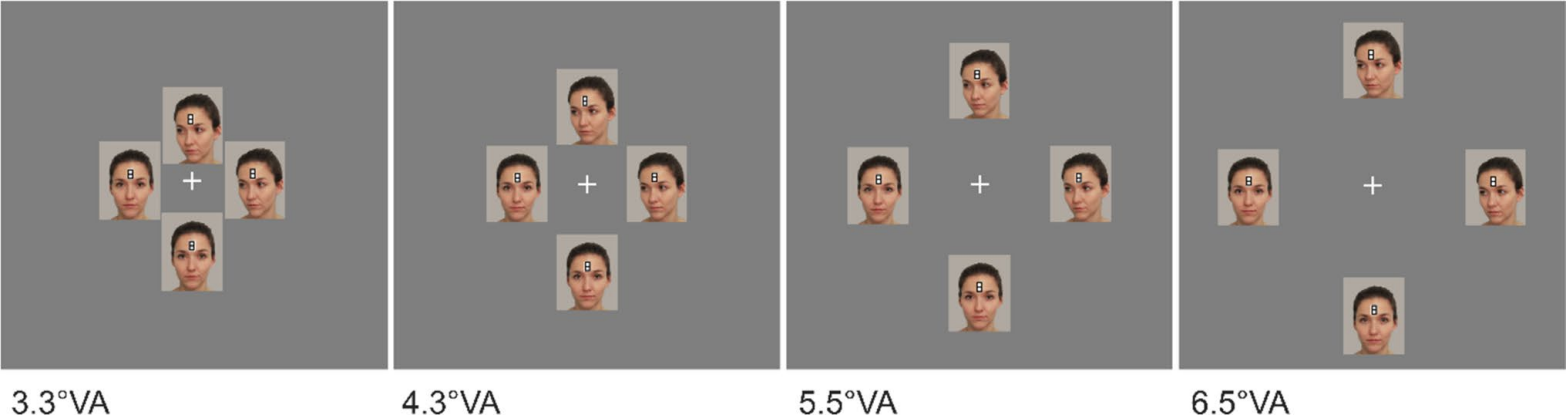
Eccentricities of stimuli relative to center in groups D1-D4. *Note.* The original eccentricity of 4.3°VA from Böckler et al. (2014) was applied to group D2 and decreased by 25% for group D1 (3.3°VA) or increased by 25% or 50% for group D3 (5.5°VA) and group D4 (6.5°VA), respectively. Please note that the figure is an approximate depiction and does not show the size of face stimuli in relation to the real screen size. All parts of this figure are original. Photographs were taken by the authors and printed with permission

To sum up, previous research suggests that effects of eccentricity on object processing are greatly determined by the type of object, the task at hand, and the object features to be processed. However, a systematic assessment of eccentricity effects on the processing of face motion and direction within a single comprehensive study is still lacking. Addressing this matter, we applied the paradigm of Böckler et al. (2014, 2015, see Fig. 1) and systematically varied the eccentricity of the face stimuli between groups of participants. In particular, we conceptually replicated the experiments with the original eccentricity of 4.3° VA (group D2). Furthermore, we either moved the stimuli closer to the central fixation cross (group D1: −25%, 3.3°VA) or further away from it (group D3: +25%, 5.5°VA; group D4: +50%, 6.5°VA; see Fig. 2). The key questions were whether and how the face/gaze direction effect and the sudden onset motion effect were modulated by eccentricity. In an additional control experiment, we tested whether face/gaze direction could be adequately perceived in the periphery (i.e., at 5.5° and 6.5°). To this end, we employed a direction discrimination task using the same face stimuli as in the main experiment with eye tracking to ensure central fixation throughout the task.

## Methods

### Participants

A total of 165 participants were randomly assigned to one of the four groups of the main experiment^1^. We removed the data sets of 5 participants due to excessively high error rates (ER > 2 SD of the global within-group error rate). The final sample of the main experiment thus comprised 160 participants. Sensitivity analyses in G*Power 3.1 (Faul et al., 2007) showed that with this sample size, an effect size of f=0.11 or η^2^≥0.012 can be detected with a statistical power of 1-β=.80 in an ANOVA with an alpha-level of .05. This effect size is comparably smaller than those revealed for face/gaze direction and motion onset effects in previous studies (e.g., in Böckler et al., 2014). For the control experiment, we recruited 19 participants, of which one had to be excluded due to an incomplete data set. Demographic details of the main sample (split by group) and the control sample as well as participant exclusions are displayed in Table 1. All participants gave written informed consent prior to testing and were compensated monetarily or with course credit. The present study is compliant with the ethical standards of the 1964 Declaration of Helsinki regarding the treatment of human participants and was approved by the local ethics committee.

**Table 1:** Demographic details of main sample (split by group) and control sample

Sample	Total *N* (Final *N*)	Mean age (SD) years	Percentage (%) of females	Number of right-handed participants	Excluded due to mean error rate > 2 SD
D1: 3.3°	42 (41)	24.8 (4.5)	73.2	35	1
D2: 4.3°	41 (39)	24.2 (4.4)	82.1	36	2
D3: 5.5°	40 (40)	24.1 (4.1)	72.5	35	0
D4: 6.5°	42 (40)	26.7 (4.9)	70	33	2
Control	18 (18)	24.2 (4.9)	83.3	17	0

*Note*. Final *N* = number of participants after data exclusion. Mean age, percentage of females (all participants of the current study identified as either female or male) and number of righthanded participants after data exclusion

### Material and procedure

In the main experiment, we employed the paradigm developed by Böckler et al. (2014, 2015) and varied the distance of the face-stimuli from the central fixation cross between groups. Participants were placed 80 cm away from a 69-cm diagonal (59.77cm × 33.62cm) monitor (screen resolution: 81.6 dpi; 1920 × 1080 pixels) with their two index fingers placed on the response keys (“S” and “H”) of a standard German QWERTZ keyboard. Each trial began with four images of the same female face (each face 139 × 171 pixels, 3 × 3.7°VA), two directed towards the onlooker (with direct face and gaze) and two with their face/gaze averted by 45° to the bottom left, located around a central fixation cross (see Fig. 1). Small figure-8 symbols (0.3 × 0.5°VA) were positioned on the forehead of each face (2.2 × 3.4°VA) and replaced by one target (“S”/”H”) and three distractor letters (“E”/”U”) after 1500 ms. Simultaneously with target presentation, one direct face changed to averted and one averted face changed to direct (inducing apparent motion, e.g., Wertheimer, 1912) while the other two faces remained unchanged (no motion). The directions of the direct and averted faces were the same at all four positions and the extent of motion was similar for sudden-direct and sudden-averted faces to minimize differences between stimuli beyond face/gaze direction and motion condition. Each participant went through a total of 384 trials, with 96 trials per face/gaze direction and motion condition. The identity and position of target and distraction letters appeared equally often in all possible combinations. Prior to the task and during training trials, participants were explicitly instructed to fixate on the central cross throughout the experiment and to respond as quickly and accurately as possible to the target letter by pressing the respective response key. Note that we did not employ eye-tracking measures to verify constant fixation of the screen center. Hence, paying attention to motion or face/gaze direction was not necessary for task completion. Importantly, while the factors motion and face/gaze direction were varied randomly within participants, the distance of the stimuli to the central fixation was kept constant for each participant and was manipulated between groups of participants (groups D1 - D4). This was done to reduce fatigue-related effects of prolonged experimental sessions that might have resulted in a loss of power to detect any modulation of the gaze/face direction effect and/or the sudden motion onset effect by stimulus presentation eccentricity. In group D2, the distance from the inner edge of the target letter to the center of the screen was the same as in the previous studies (4.3° VA, see Böckler et al., 2014, 2015; Breil, Huestegge et al., 2022a, Breil, Raettig et al., 2022b). Relative to D2, the distance was decreased by 25% (3.3° VA) in group D1, increased by 25% (5.5° VA) in group D3, and increased by 50% (6.5° VA) in group D4 (see Fig. 2). PsychoPy v1.83.01 (Peirce et al., 2019) was used for stimulus presentation and response recording.

An additional control experiment employed eye tracking to test whether face/gaze direction could accurately be perceived using peripheral vision. In this task, a single cue stimulus from the main experiment (with number-8 figures on the forehead) was presented at eccentricities of 5.5. or 6.5°VA at a random position to the top, bottom, left, or right of the central fixation cross. Participants were instructed to fixate the central fixation cross throughout a trial and judge the direction of face/gaze (direct or averted) by pressing a corresponding key on a CEDRUS button box. Importantly, trials started only after a fixation on the central cross was registered and were immediately terminated if a surrounding rectangular area of interest (AOI, 150 x 150 pixels, 3.3 x 3.3° VA) was left. Fixation errors were followed by a 1000 ms long error message prompting the participant to re-fixate the central cross, after which the same trial was repeated. All trials containing such a fixation error were discarded from statistical analyses. After 20 training trials, 320 test trials were randomly presented in two blocks with a break in between. Eye movements were recorded with an EyeLink 1000 Plus eye tracking system (SR Research Mississauga, Ontario, Canada) with a sampling rate of 1000 Hz, and the corresponding software (Experiment Builder; Data Viewer) was implemented for stimulus presentation and data preprocessing.

### Data analysis

We defined reaction time (RT) as the time window from target onset until key press. Error rates (%) were calculated from all trials for each participant in each experimental cell. Error trials as well as outliers (RT exceeding ±2 SD of the participant’s cell mean) were removed from RT analyses (see Table 2). Using more liberal criterion for RT trimming (± 3 SD of the cell mean) and excluding RT-trimmed trials from ER analyses did not substantially change the pattern of results (see Tables S1 and S2 in the supplemental material). The remaining trials were used to compute each participant’s mean RT in each experimental cell. The face/gaze direction advantages in RTs and ERs were calculated for each stimulus eccentricity by subtracting direct face/gaze RTs (ERs) from averted face/gaze RTs (ERs), averaged across motion condition. Motion onset advantages were calculated by subtracting sudden motion RTs (ERs) from static RTs (ERs), averaged across face/gaze directions. Positive values therefore indicate a relative advantage of direct face/gaze and motion onset. Raw RTs and ERs were analyzed with separate 2x2x4 mixed ANOVAs with the within-subject factors face/gaze direction (direct, averted) and motion (static, sudden) and the between-subjects factor eccentricity (D1: 3.3°, D2: 4.3°, D3: 5.5°, D4: 6.5°). Note that the factor face/gaze direction refers to the orientation of the target face in the target/distractor display, not to the orientation of the same face in the cue display. Significant interaction effects were followed up with simple main effect analyses or pairwise comparisons of the estimated marginal means. Data analysis was conducted using R (R Core Team, 2023), and the papaja package (Aust & Barth, 2014/2022) was used to write computationally reproducible methods and results sections. The tidyverse package (Wickham et al., 2019) was used for general data processing. The afex package (Singmann et al., 2023) and the emmeans package (Lenth et al., 2023) were used for conducting frequentist ANOVAs and follow-up analyses, respectively. To quantify evidence for the absence of effects, we additionally conducted Bayesian ANOVAs using the BayesFactor package (Morey et al., 2022) with a Cauchy scale parameter of 1. We calculated Bayes factors (BF01) for each non-significant main effect and interaction term by comparing the model without the respective effect in the enumerator against the full model in the denominator. We interpreted BF01 > 3 as evidence for the absence of an effect.

**Table 2:** Total number of trials, number of errors, number of outliers (+/- 2 SD beyond the mean), and number of trial exclusions in the main experiment as a function of eccentricity (group)

Eccentricity	Trials (n)	Errors (n)	Errors (%)	Outliers (n)	Outliers (%)	Excluded (n)	Excluded (%)
D1: 3.3°	15744	402	2.55	621	3.94	1023	6.50
D2: 4.3°	14976	590	3.94	533	3.56	1123	7.50
D3: 5.5°	15360	547	3.56	473	3.08	1020	6.64
D4: 6.5°	15360	534	3.48	464	3.02	998	6.50

*Note.* Total trial counts differ between groups because of the slightly unequal sample sizes after participant exclusion

In the control experiment, trials with a fixation error (i.e., an offset of central fixation was detected) were discarded from all analyses (15.90 % of all trials). From RT analyses, we further excluded errors (6.96 % of all trials) and outliers (4.53 % of all trials). RTs and error rates were submitted to two 2×2 repeated measures ANOVAs with the within-subject factors face/gaze direction (direct, averted) and eccentricity (5.5, 6.5°VA). Bayesian ANOVAs were conducted and interpreted as described above.

## Results

**Fig. 3:**
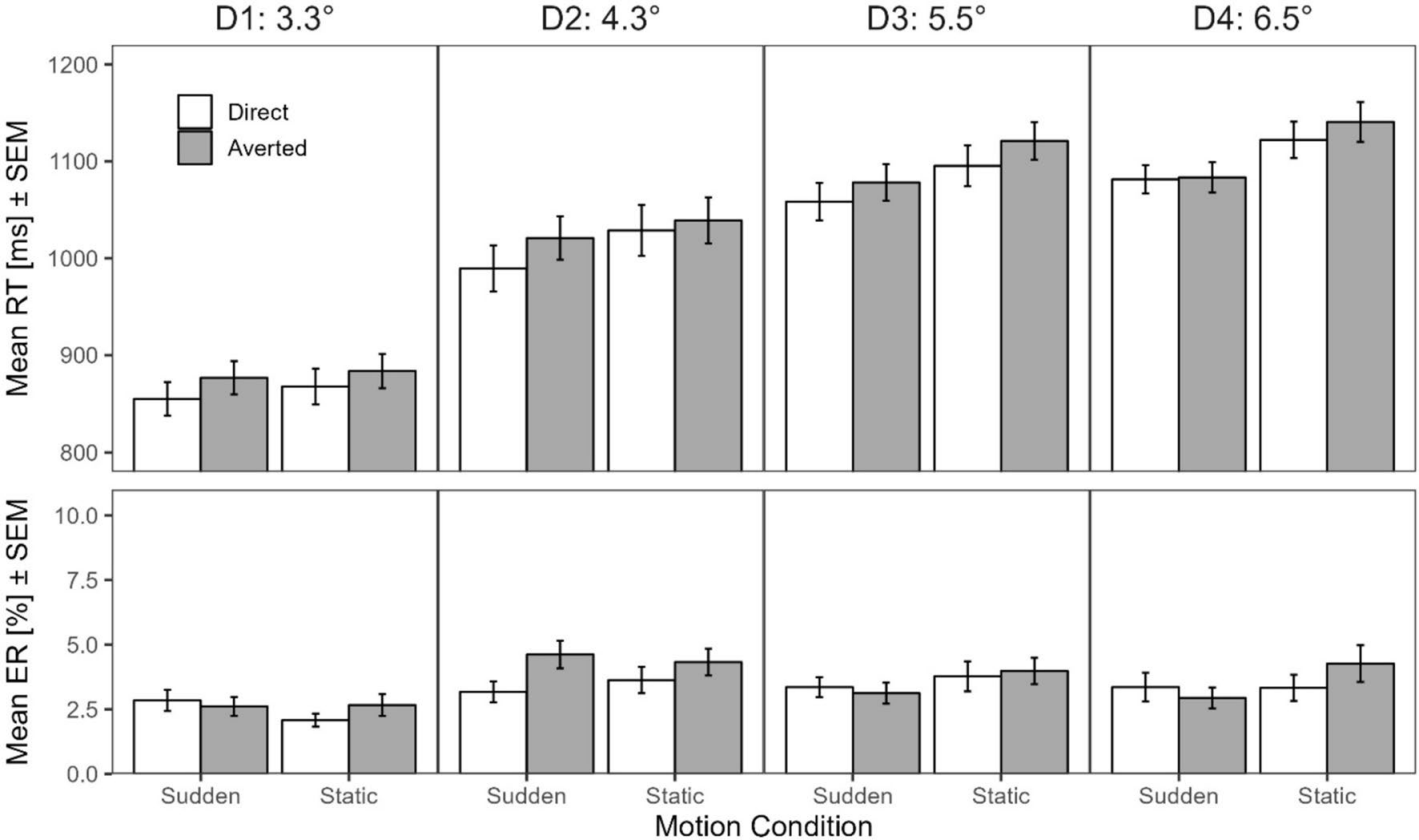
Results of main experiment. *Note*. Mean RT and ER as a function of stimulus eccentricity (in °VA , manipulated across groups, separate panels), motion condition (sudden direction shift vs. static direction, manipulated within-subject, x-axis), and faze/gaze direction (direct vs. averted, manipulated within-subject, white and grey bars, respectively). Error bars represent standard errors of the mean (SEM)

**Fig. 4:**
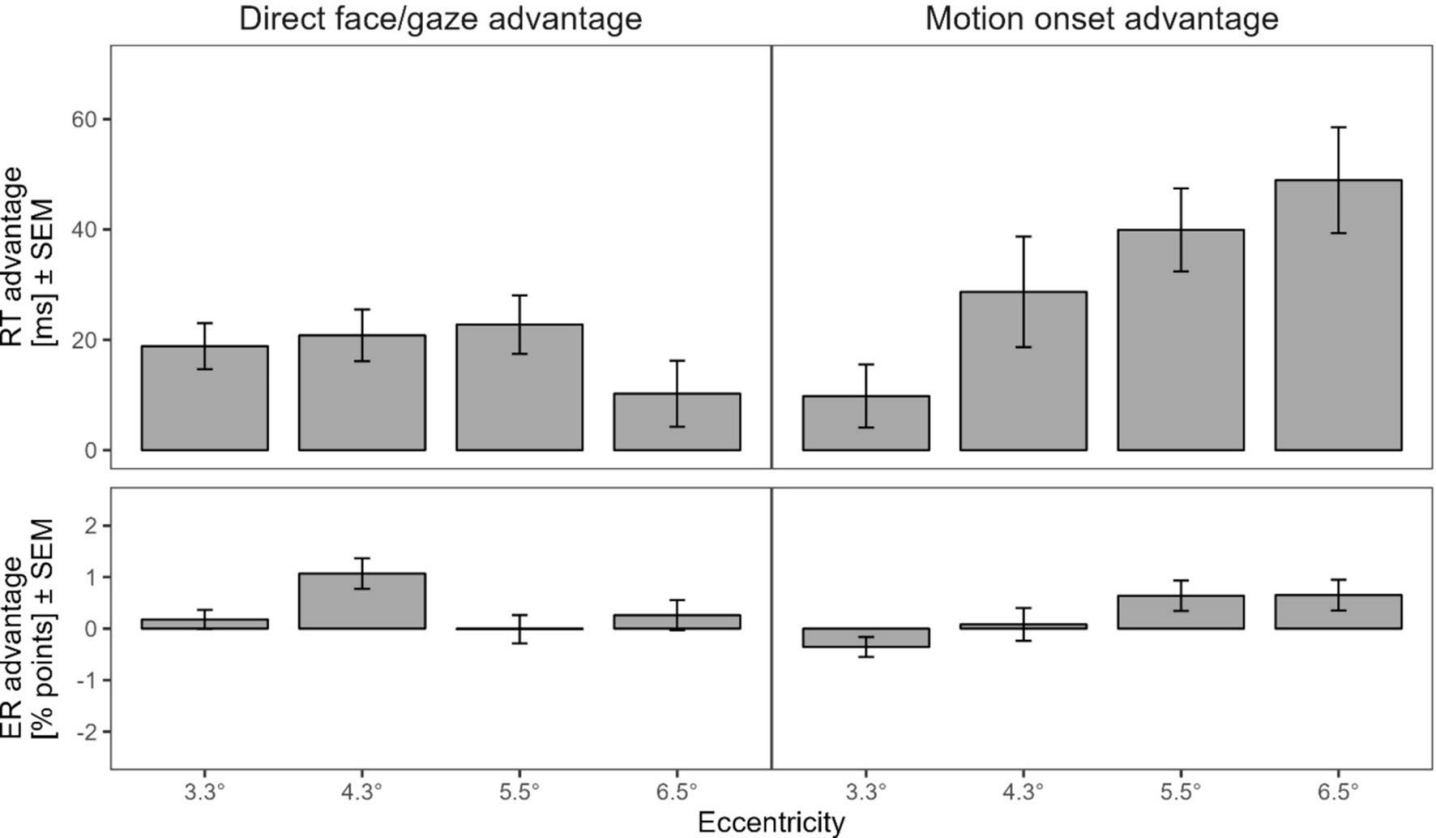
Direct-gaze and motion-onset advantages. *Note*. RT advantages (in ms) and ER advantages (in % points) of direct face/gaze and sudden onset motion as a function of stimulus eccentricity. Face/gaze direction advantages were calculated by subtracting direct face/gaze RTs (ERs) from averted face/gaze RTs (ERs), averaged across motion condition. Motion onset advantages were calculated by subtracting sudden motion RTs (ERs) from static RTs (ERs), averaged across face/gaze directions. Positive values therefore indicate a relative advantage of direct gaze / motion onset. Error bars represent standard errors of the mean (SEM)

Table 3 contains descriptive summary statistics of the main experiment, Table 4 contains descriptive summary statistics of the control experiment. Figure 3 depicts mean RTs and ERs for each condition of the main experiment. Figure 4 visualizes the face/gaze direction effect and sudden onset motion effect as a function of eccentricity, calculated as the mean RT (ER) for direct minus for averted stimuli and the mean RT (ER) for sudden minus for static stimuli, respectively.

**Table 3:** Descriptive summary statistics for the main experiment

Eccentricity	Condition							
	Sudden Direct		Static Direct		Sudden Averted		Static Averted	
	RT (SE)	ER (SE)	RT (SE)	ER (SE)	RT (SE)	ER (SE)	RT (SE)	ER (SE)
D1: 3.3°	855 (17)	2.84 (0.41)	868 (18)	2.08 (0.26)	877 (17)	2.62 (0.36)	884 (18)	2.67 (0.42)
D2: 4.3°	990 (24)	3.18 (0.40)	1029 (26)	3.63 (0.51)	1021 (22)	4.62 (0.53)	1039 (24)	4.33 (0.52)
D3: 5.5°	1058 (19)	3.36 (0.39)	1095 (21)	3.78 (0.58)	1078 (19)	3.12 (0.41)	1121 (19)	3.98 (0.51)
D4: 6.5°	1081 (15)	3.36 (0.56)	1122 (19)	3.33 (0.51)	1083 (16)	2.94 (0.40)	1141 (20)	4.27 (0.71)

*Note.* Mean (SE in parentheses) of correct RTs (ms) and ERs (%) as a function of eccentricity (group), motion and gaze

**Table 4:** Descriptive summary statistics for the control experiment

Eccentricity	Face/gaze direction	RT (SE)	ER (SE)
5.5°	Direct	569 (34)	5.10 (1.88)
6.5°	Direct	568 (32)	7.05 (2.48)
5.5°	Averted	585 (32)	8.70 (3.13)
6.5°	Averted	592 (32)	9.38 (3.31)

*Note.* Mean (SE in parentheses) of correct RTs (ms) and ERs (%) as a function of eccentricity and gaze

### Main experiment

#### RT data

We found a significant main effect of eccentricity, $F\left(3,156\right)=32.12$, $p<.001$, ${\widehat{\eta }}_{p}^{2}=.382$, due to increasing RTs with increasing eccentricity. On average, participants responded after 871 ms (*SD* = 113 ms) in group D1, after 1020 ms (*SD* = 150 ms) in group D2, after 1,088 ms (*SD* = 126 ms) in group D3 and after 1107 ms (*SD* = 113 ms) in group D4. The main effect of face/gaze direction was significant, $F\left(1,156\right)=51.02$, $p<.001$, ${\widehat{\eta }}_{p}^{2}=.246$, due to an overall direct gaze advantage. The main effect of motion was significant as well, $F\left(1,156\right)=58.37$, $p<.001$, ${\widehat{\eta }}_{p}^{2}=.272$, indicating an overall motion-onset advantage. Critically, there was a significant two-way interaction between motion and eccentricity, $F\left(3,156\right)=4.15$, $p=.007$, ${\widehat{\eta }}_{p}^{2}=.074$. Simple main effect analyses by distance revealed that the motion-onset advantage increased from a non-significant 10 ms (*SD* = 37 ms, $F\left(1,156\right)=1.42$, $p=.235$) in group D1 over 29 ms (*SD* = 63 ms, $F\left(1,156\right)=11.56$, $p=.001$) in group D2 and 40 ms (*SD* = 48 ms, $F\left(1,156\right)=22.95$, $p<.001$) in group D3 to 49 ms (*SD* = 61 ms, $F\left(1,156\right)=34.46$, $p<.001$) in group D4. In contrast, there was no significant interaction between face/gaze direction and eccentricity, $F\left(3,156\right)=1.18$, $p=.321$, ${\widehat{\eta }}_{p}^{2}=.022$, ${\text{BF}}_{01}=1.54\times {10}^{2}$, indicating that the direct face/gaze advantage remained relatively stable across eccentricity groups. This advantage amounted to 19 ms (*SD* = 27 ms, $F\left(1,156\right)=14.10$, $p<.001$) in group D1, to 21 ms (*SD* = 29 ms, $F\left(1,156\right)=16.32$, $p<.001$) in group D2, to 23 ms (*SD* = 34 ms, $F\left(1,156\right)=20.03$, $p<.001$) in group D3, and to 10 ms (*SD* = 38 ms, $F\left(1,156\right)=4.05$, $p=.046$) in group D4. We also found a significant three-way interaction between face/gaze direction, motion and eccentricity $F\left(3,156\right)=2.74$, $p=.045$, ${\widehat{\eta }}_{p}^{2}=.050$. This was mainly due to the significant two-way interaction between gaze and motion in group D2, $F\left(1,156\right)=4.61$, $p=.033$. Here, participants, responded faster to stimuli on direct-gaze faces when the same face also showed a sudden onset motion $, t\left(156\right)=-4.71$, $p<.001$, but not when the same face was static, $t\left(156\right)=-1.36$, $p=.175$. The same interaction was non-significant in all other groups (all *F*s < 2.92, all *p*s > .089). Overall, there was no significant two-way interaction between face/gaze direction and motion, $F\left(1,156\right)=0.05$, $p=.819$, ${\widehat{\eta }}_{p}^{2}=.000$, ${\text{BF}}_{01}=1.54\times {10}^{1}$.

#### Error data

We found a significant main effect of face/gaze direction, $F\left(1,156\right)=7.91$, $p=.006$, ${\widehat{\eta }}_{p}^{2}=.048$. The slight direct face/gaze advantage suggests that the main effect of face/gaze direction in RTs was not due to a speed-accuracy trade-off. No other main effect was significant (both *F*s < 3.10, both *p*s > .070, both BF01 > 4.20). We also found a significant two-way interaction between face/gaze direction and eccentricity, $F\left(3,156\right)=3.17$, $p=.026$, ${\widehat{\eta }}_{p}^{2}=.057$. Simple main effect analyses revealed that the direct face/gaze advantage was not significant in groups D1 ($F\left(1,156\right)=0.46$, $p=.499$), D3 ($F\left(1,156\right)=0.00$, $p=.964$) and D4 ($F\left(1,156\right)=0.96$, $p=.328$) while it was significant in group D2 ($F\left(1,156\right)=15.77$, $p<.001$). The two-way interaction between motion and distance was significant as well, $F\left(3,156\right)=3.06$, $p=.030$, ${\widehat{\eta }}_{p}^{2}=.056$. Simple main effect analyses revealed that the motion effect was not significant in groups D1 ($F\left(1,156\right)=1.66$, $p=.199$) and D2 ($F\left(1,156\right)=0.08$, $p=.775$) while it was significant in groups D3 ($F\left(1,156\right)=5.22$, $p=.024$) and D4 ($F\left(1,156\right)=5.44$, $p=.021$). Importantly, this interaction points in the same direction as in the RT data, indicating that RT effects were not compromised by a speed-accuracy trade-off. No other interaction reached significance (both *F*s < 2.29, both *p*s > .080, both BF01 > 5.07).

### Control experiment

In the RT data, we found a significant main effect of face/gaze direction, $F\left(1,17\right)=7.57$, $p=.014$, ${\widehat{\eta }}_{p}^{2}=.308$ due to faster recognition of direct compared to averted stimuli. Neither the main effect of distance ($F\left(1,17\right)=0.40$, $p=.535$, ${\widehat{\eta }}_{p}^{2}=.023$, ${\text{BF}}_{01}=6.30$) nor the interaction between face/gaze direction and distance ($F\left(1,17\right)=1.09$, $p=.310$, ${\widehat{\eta }}_{p}^{2}=.060$, ${\text{BF}}_{01}=3.78$) reached significance. These results were mirrored by the error data. Participants made less errors in response to direct-gaze faces than in response to averted-gaze faces, $F\left(1,17\right)=5.12$, $p=.037$, ${\widehat{\eta }}_{p}^{2}=.231$. Again, there was no significant main effect of distance ($F\left(1,17\right)=1.24$, $p=.282$, ${\widehat{\eta }}_{p}^{2}=.068$, ${\text{BF}}_{01}=3.47$) and no significant interaction ($F\left(1,17\right)=0.90$, $p=.356$, ${\widehat{\eta }}_{p}^{2}=.050$, ${\text{BF}}_{01}=4.33$). Critically, overall low error rates and reasonably fast responses suggest clear recognizability of gaze direction also in the more extreme eccentricity conditions of our main experiment.

## Discussion

The present study systematically manipulated presentation eccentricity of human faces and addressed its effect on attention capture by direct face/gaze and sudden onset motion. In four groups of participants (total *N*=160), photographic faces were presented at different distances to a central fixation, spanning 3.3°, 4.3°, 5.5° and 6.5° of the visual field, while physical stimulus size was held constant. Across all direction and motion conditions, we found a processing benefit for stimuli that were presented more centrally. This finding of a general distance effect is in line with previous studies (e.g. Carrasco et al., 1998; Gruber et al., 2014) and serves as an important manipulation check. In addition, we replicated the finding that social attention towards the participant and sudden onset motion presented at 4.3° eccentricity capture attention (Böckler et al., 2014; Boyer & Wang, 2018; Breil, Huestegge et al., 2022a).

Critically, the systematic manipulation of eccentricity caused markedly differential modulations of the face/gaze direction effect and the sudden onset motion effect. We found that the direct face/gaze benefit was not significantly modulated by distance and did not change in size when the original distance was varied by ±25% (3.3°, group D1; 5.5°, group D3, respectively), providing further evidence for the effect’s generalizability. However, while this effect was relatively stable in magnitude (about 20 ms) until 5.5° of eccentricity, it was numerically cut in half (about 10 ms), albeit still significant, at 6.5° (group D4). Importantly, our control experiment shows that face/gaze direction could still be reliably discriminated at the higher eccentricities. This pattern is consistent with research that find gaze cueing effects when the stimulus is presented at 5.0°, but no longer at 7.5° (Yokoyama & Takeda, 2019) and that judgements of gaze direction are reliable up to 4 or 5° when the head remains static (Loomis et al., 2008; Palanica & Itier, 2017). Beyond an angle of 5.5°, direct face/gaze appears to be less salient, but still salient enough to capture attention, as long as it can be perceived with sufficient precision. Hence, basic social signals like a direct gaze exert a relatively consistent influence on our attentional system in the close periphery, with a potential reduction only at farther distances.

In contrast, the processing benefit for sudden onset motion significantly *increased* with eccentricity. While attention capture by motion was absent at the smallest eccentricity (3.3°; Group D1), it was statistically significant and numerically increased with increasing distance from fixation, confirming the finding of a higher motion sensitivity in the periphery (Burnat, 2015; Finlay, 1982; Kowler, 2011). The efficient detection of sudden movements in the periphery is an important mechanism to redirect foveal attention that could help to facilitate quick reactions to objects and events in the environment (Burnat, 2015; Kowler, 2011). This notion has been substantiated by the finding that motion-sensitive areas of the dorsal stream respond to attentional enhancement in the periphery (Bressler et al., 2013) and that the peripheral visual field is specifically tuned to high velocities (Kitterle, 1986).

This overall pattern of differential modulation of face/gaze direction and motion effects by eccentricity replicates and extends previous research suggesting that attention capture by direct gaze and by sudden onset motion are indeed based on different processing channels (Böckler et al., 2014). The effect of eccentricity on the sudden onset motion benefit corresponds with the dorsal (“where”) channel of visual processing that allows efficient processing even and especially in the visual periphery (Goodale & Milner, 1992). The dorsal stream is particularly involved in action and object location and comprises areas that are important for subsequent eye position and attention shifts (Bressler et al., 2013; Corbetta & Shulman, 2002). In contrast, the observed (null-) effect of eccentricity on direct face/gaze processing is not as clearly attributable to processing in the ventral (“what”) channel. Ventral stream processing is usually restricted to the (para-)foveal regions of the visual field due to the dependence on high visual acuity for most object identification. Instead, the relative robustness of attention capture by direct face/gaze in the current study, in line with other findings of preserved face/gaze discrimination in the periphery (Hershler et al., 2010; Jebara et al., 2009; Loomis et al., 2008; Palanica & Itier, 2017), might be taken as support for recent suggestions of a separate pathway on the lateral brain surface dedicated to processing (usually dynamic) social signals (Pitcher & Ungerleider, 2021). This so called “social pathway” might equally weigh input from more foveal and peripheral visual areas. Our present findings fit this line of research by showing that attention capture by direct face/gaze and motion cues are differentially modulated by eccentricity. While we attend to the direction of another’s orientation with both central and (close) peripheral vision, reacting to sudden onset motion becomes a priority in the periphery. In the following, we will discuss some aspects of our results in more detail.

In the present study, head orientation always corresponded with gaze direction. In contrast to gaze direction, head orientation seems to be accurately perceived at much higher eccentricities (Loomis et al., 2008). There is evidence that from ±3° eccentricity, head orientation starts to increasingly bias perceptions of gaze direction (Palanica & Itier, 2014). Specifically, head orientation seems to be used as a (valid) cue for gaze direction, which might be one reason for the preservation of the direct-gaze effect in our study. This relatively stable processing benefit for direct gaze differs from Palanica and Itier’s (2014) finding of a *gradual* decrease of gaze direction discrimination performance with increasing eccentricity of the face stimuli. Future studies should therefore independently manipulate gaze direction and head orientation to investigate the modulation of the direct-gaze effect by head orientation, ideally at different eccentricities.

Another aspect that could be addressed in future studies involves the central-to-peripheral compression of projections from the retina to the striate cortex (cortical magnification factor). To unequivocally assess the influence of eccentricity on the effects of face/gaze direction and motion, we held the angular size of stimuli constant across all experiments of this study. However, perceptual acuity decreases from central to peripheral vision and the area of the visual cortex that is filled by a stimulus gets smaller the further away from the fovea it is presented (Anstis, 1998). Hence, constant visual acuity can be achieved by increasing the target size according to perceptual eccentricity (Anstis, 1974). We found, in our study with constant physical stimulus size, that attention capture by direct face/gaze was relatively invariant across the central-to-peripheral gradient of the visual field while attention capture by sudden onset motion numerically increased. Future studies could investigate whether increased stimulus size can compensate for large eccentricity. For example, one could explore whether the face/gaze direction effect can be preserved even in the farther periphery by parametrically manipulating the angular size of stimuli according to the distance to the center.

In our study, sudden onset motion did not capture attention for stimuli presented almost centrally (3.3°). On first glance, this appears inconsistent with findings of better or equal motion detection in central compared to peripheral visual regions (Finlay, 1982; McKee & Nakayama, 1984; To et al., 2011) However, the task in the present study was not to discriminate sudden-motion-onset faced from static faces (motion detection) but to classify stimuli presented on faces that varied regarding motion and gaze direction. Even though sudden onset motion is easily discriminated in the central visual field, it might be less relevant for attentional capture. It should be noted, however, that absolute and general statements on sensitivity thresholds are not necessarily conclusive. Previous research suggests an intricate modulation of foveal and peripheral sensitivity to motion task requirements and stimulus parameters such as luminance, contrast, size and velocity (for an overview, see Sekuler et al., 2002). Although the present study provides promising initial findings, future studies are needed to systematically assess various stimulus materials at a broad range of eccentricities.

Unexpectedly, we found a significant three-way interaction in response times which was driven by a significant interaction of face/gaze direction and motion at 4.3° eccentricity that was absent in the other eccentricity groups. Responses to sudden direct stimuli were faster compared to sudden averted stimuli, whereas no significant difference was found for static stimuli. This finding contradicts earlier experiments that have not reported interaction effects between gaze and motion cues at 4.3° (Böckler et al., 2014; Boyer & Wang, 2018). The origin of this inconsistency cannot be fully explained with the present data. Given that no interaction between gaze and motion was found in any of the other groups, we tend to assume that these results reflect a random, unsystematic fluctuation in the data that we do not want to overinterpret at this point. This notion is supported by the Bayesian analysis of variance, providing support for the absence of a strong three-way interaction (BF_01_ > 20).

One might naturally question whether the effect of directed social attention observed here, as well as in numerous other studies (Böckler et al., 2014; Boyer & Wang, 2018; Leal Rato et al., 2019; Lyyra et al., 2018; Senju et al., 2005), is driven by high-level “social” information in the direct face/gaze images or by lower-level perceptual feature differences compared to the averted face/gaze images. Of course, apart from face and gaze direction, the real-life pictures on which the target letters were presented necessarily differed in several basic characteristics, such as symmetry, edge and surface features, luminance, and other low-level attributes that could affect attentional processing (Maunsell & Treue, 2006; Olivers & Van Der Helm, 1998). One possible approach to answering this question would be to invert the face images. Böckler et al. (2015), using a highly similar setup to the current one, demonstrated that attentional capture by direct gaze/face is indeed absent when using inverted face pictures. This was interpreted in terms of less holistic processing of inverted compared to upright faces. Given that some basic differences between direct and averted faces remain in inverted images (e.g., symmetry), this may suggest the contribution of higher-level social attention mechanisms (beyond low-level perceptual ones). Of course, inverted faces lack some basic features altogether (e.g., they are not top-heavy). A more rigorous approach would involve creating new stimuli based on the direct and averted face images used here and systematically manipulating their low-level features to determine precisely *why* a realistic direct gaze tends to attract attention more readily than an averted gaze. In a comparable attempt to unravel the mechanisms behind newborns’ face preference, Cassia and colleagues (2004) manipulated the up-down asymmetry of stimuli based on naturalistic face pictures by re-arranging their inner elements (eyes, mouth, nose) creating top-heavy (face-like) and bottom-heavy (non-face-like) distributions of visual information. Their findings revealed similar viewing preferences for upright face pictures and top-heavy scrambled pictures indicating that this low-level feature was a driving factor instead of a hard-wired face preference. However, the relationship between low-level features and social information in face perception is complex. Some studies have demonstrated preferential processing of social features over purely saliency-based, low-level features (e.g., Flechsenhar & Gamer, 2017). Wang and colleagues (2014) found reduced visual search performance in patients with Autism Spectrum Disorder (ASD) for social targets compared to non-social targets, even when controlling for low-level saliency differences. This suggests a difference in processing social information that cannot be explained by low-level visual features alone. Furthermore, neurophysiological evidence supports the existence of specialized neural mechanisms for social attention. Perrett et al. (1992) identified brain cells in the temporal cortex specifically tuned to different directions of social attention cues such as gaze, head and body direction. This suggests that the processing of directional social information, particularly gaze, might be fundamental in attracting and guiding attention in ways that are not reducible to low-level saliency differences. A definitive conclusion on this matter is beyond the scope of the present study, which focused on the (potentially differential) modulation of the well-documented processing benefits associated with directed social attention and sudden-motion onset as a function of stimulus eccentricity. Nevertheless, disentangling high-level social and semantic contributions from lower-level perceptual influences on the face/gaze direction effect represents an important and compelling direction for future research.

Furthermore, in addition to *capture* of attention by directed social attention, the employed setup might, in principle also induce a *diversion* of attention by averted social attention (i.e., gaze cueing, see Frischen et al., 2007; McKay et al., 2021 for reviews). Particularly, the stimuli on the left position and on the bottom position might be cued by averted faces in the top or right location, respectively (see Figure S1 in the supplemental material). Indeed, when considering only trials with the target letter present on the left or bottom position, we found an RT (but no ER) advantage for averted (vs. direct) upper-diagonal stimuli (i.e., a gaze-cueing effect, see Table S4 and Figure S2 in the supplemental material) which was not significantly modulated by eccentricity. To rule out the possibility that the effects of attention *capture* found in the main analyses of this study were explained by this *diversion* of attention, we conducted the original analyses excluding trials with the target letter on the left or bottom position which did not change the qualitative pattern of results (see Table S5 and Figure S3 in the supplemental material). Nevertheless, gaze cueing by peripheral face stimuli seems to operate in addition to attention capture by face/gaze direction and sudden-onset motion and should thus be investigated more closely in future studies on social attention.

## Conclusion

Our results corroborate and extend previous findings regarding attention capture by realistic face/gaze and motion cues in the periphery (Böckler et al., 2014, 2015; Boyer & Wang, 2018; Breil, Huestegge et al., 2022a, Breil, Raettig et al., 2022b). Specifically, our data suggest eccentricity-independent attention capture by direct face/gaze as long as face/gaze direction can be discriminated. If anything, the face/gaze direction effect numerically decreased in the farther visual periphery. By contrast, processing benefits for sudden onset motion systematically increased with eccentricity while being absent for near central stimuli. On a more general level, our results emphasize the importance of taking specific stimulus properties into account when studying perception and attention in the periphery. Finally, the current study highlights the sophisticated adaptation of the human attentional and visual system, allowing us to navigate our (social) environment flexibly and efficiently.

## Supplementary Information

Below is the link to the electronic supplementary material.

Supplementary file1 (DOCX 361 KB)

## Data Availability

The data availability is covered in the Open practices statement. A link to the repository containing data and analysis scripts is provided there.

## Footnotes

1 Note that half of the main sample was collected between 06/2019, and 11/2019, while the other half was collected between 02/2023, and 07/2023, under near identical conditions.
