# Supplementary material for: How eccentricity modulates attention capture by direct face/gaze and sudden onset motion
Source: Atten Percept Psychophys. 2025 Feb 6;87(2):354–66. doi: 10.3758/s13414-025-03015-8 (PMC11865121; doi:10.3758/s13414-025-03015-8)
Supplement: Supplementary file 1 — Supplementary file1 (DOCX 361 KB) [file 13414_2025_3015_MOESM1_ESM.docx]

Supplemental material to: How eccentricity modulates attention capture by direct face/gaze and sudden onset motion

Jens Kürten^1*^, Christina Breil^1,2*^, Roxana Pittig^1,3^, Lynn Huestegge^1^, & Anne Böckler^1^

^1^ University of Würzburg

^2^ Institut für Gesundheitsförderung und Prävention, Vienna

^3^ Georg-Elias-Müller-Institut für Psychologie, Universität Göttingen

* These authors contributed equally to this work.

Author note

Jens Kürten*, Institute of Psychology, University of Würzburg; Christina Breil*, Institute of Psychology, University of Würzburg, Institut für Gesundheitsförderung und Prävention, Vienna; Roxana Pittig, Institute of Psychology, University of Würzburg, Georg-Elias-Müller-Institut für Psychologie, Universität Göttingen; Lynn Huestegge, Institute of Psychology, University of Würzburg; Anne Böckler, Institute of Psychology; University of Würzburg. Raw data and analysis scripts are available at (<https://osf.io/w9zgh/?view_only=61a26da938a0440f9ce04c424602779b>).

* These authors contributed equally to this work.

The authors have no commercial relationships and no competing interests to declare. The present study was supported by the German Research Foundation (Grant numbers BO 4962/1-1 & HU 1847/9-1).

Correspondence concerning this article should be addressed to Jens Kürten, Röntgenring 11, 97070 Würzburg, Germany. E-mail: jens.kuerten@uni-wuerzburg.de

Abstract

We investigated how processing benefits for direct face/gaze and sudden onset motion depend on stimulus presentation location, specifically eccentricity from fixation. Participants responded to targets that were presented on one of four stimuli that displayed a direct or averted face and gaze either statically or suddenly. Between participants, stimuli were presented at different eccentricities relative to central fixation, spanning 3.3°, 4.3°, 5.5° or 6.5° of the visual field. Replicating previous studies, we found processing advantages for direct (vs. averted) face/gaze and motion onset (vs. static stimuli). Critically, while the motion-onset advantage increased with increasing distance to the center, the direction was not significantly modulated by target eccentricity. Results from a control experiment with eye tracking indicate that face/gaze direction could be accurately discriminated even at the largest eccentricity. These findings demonstrate a distinction between the processing of basic facial and gaze signals and exogenous motion cues, which may be based on functional differences between central and peripheral retinal regions. Moreover, the results highlight the importance of taking specific stimulus properties into account when studying perception and attention in the periphery.

*Keywords:* direct face, direct gaze, motion, social attention, visual attention, spatial attention, stimulus eccentricity

Supplemental material to: How eccentricity modulates attention capture by direct face/gaze and facial motion

**Table S1***Descriptive results with alternate data trimming criteria*

| Eccentricity | Condition | | | | | | | |
| --- | --- | --- | --- | --- | --- | --- | --- | --- |
|  | Sudden Direct | | Static Direct | | Sudden Averted | | Static Averted | |
|  | RT (SE) | ER  (SE) | RT (SE) | ER  (SE) | RT (SE) | ER  (SE) | RT (SE) | ER  (SE) |
| D1: 3.3° | 870 (17) | 2.89 (0.42) | 884 (19) | 2.11 (0.26) | 895 (17) | 2.66 (0.37) | 899 (18) | 2.70 (0.42) |
| D2: 4.3° | 1011 (25) | 3.22 (0.41) | 1051 (28) | 3.67 (0.51) | 1042 (22) | 4.69 (0.54) | 1061 (25) | 4.38 (0.53) |
| D3: 5.5° | 1079 (20) | 3.39 (0.39) | 1113 (22) | 3.82 (0.59) | 1097 (19) | 3.17 (0.41) | 1138 (20) | 4.02 (0.51) |
| D4: 6.5° | 1101 (16) | 3.39 (0.57) | 1134 (19) | 3.37 (0.51) | 1104 (16) | 2.97 (0.41) | 1154 (21) | 4.30 (0.71) |

*Note.* Mean (SE in parentheses) of correct RTs (ms) and ERs (%) as a function of eccentricity (group), motion and gaze. RT means calculated with a more liberal outlier criterion than in the main experiment (± 3SD instead of ± 2SD) and ER means calculated with RT-trimmed trials removed.

**Table S2***Statistical test results with alternate data trimming criteria*

| Effect | *df* | RT | | | | ER | | | |
| --- | --- | --- | --- | --- | --- | --- | --- | --- | --- |
|  |  | *F* | *p* | $\hat{\eta}_{p}^{2}$ | BF_01_ | *F* | *p* | $\hat{\eta}_{p}^{2}$ | BF_01_ |
| Eccentricity | 3, 156 | 29.71 | < .001 | .364 | $5.32\times{10}^{-13}$ | 2.05 | .109 | .038 | 0.66 |
| Face/Gaze Direction | 1, 156 | 52.23 | < .001 | .251 | $1.41\times{10}^{-6}$ | 7.95 | .005 | .049 | 4.57 |
| Motion | 1, 156 | 52.73 | < .001 | .253 | $1.37\times{10}^{-17}$ | 3.14 | .078 | .020 | 4.78 |
| Eccentricity $\times$ Face/Gaze Direction | 3, 156 | 0.84 | .472 | .016 | $2.56\times{10}^{2}$ | 3.23 | .024 | .058 | 4.34 |
| Eccentricity $\times$ Motion | 3, 156 | 3.14 | .027 | .057 | $2.15\times{10}^{-1}$ | 3.06 | .030 | .056 | 9.34 |
| Face/Gaze Direction $\times$ Motion | 1, 156 | 0.11 | .739 | .001 | $1.58\times{10}^{1}$ | 2.41 | .123 | .015 | 5.73 |
| Eccentricity $\times$ Face/Gaze Direction $\times$ Motion | 3, 156 | 3.00 | .032 | .055 | $1.39\times{10}^{1}$ | 2.25 | .085 | .041 | 6.72 |

*Note*. ANOVA effect size estimate = $\hat{\eta}_{p}^{2}$; BF_01_ quantifies the evidence for the absence of a main effect or interaction.

**Table S3***Statistical test results of gaze cueing trials only*

| Effect | *df* | RT | | | | ER | | | |
| --- | --- | --- | --- | --- | --- | --- | --- | --- | --- |
|  |  | *F* | *p* | $\hat{\eta}_{p}^{2}$ | BF_01_ | *F* | *p* | $\hat{\eta}_{p}^{2}$ | BF_01_ |
| Eccentricity | 3, 156 | 24.63 | < .001 | .321 | $9.45\times{10}^{-11}$ | 1.29 | .280 | .024 | 11.77 |
| Gaze Cueing | 1, 156 | 16.91 | < .001 | .098 | $5.82\times{10}^{-3}$ | 1.58 | .211 | .010 | 7.25 |
| Eccentricity x Gaze Cueing | 3, 156 | 1.12 | .342 | .021 | $37.8$ | 1.51 | .213 | .028 | 20.79 |

*Note*. Only trials with the target presented at the left or bottom positions were considered for this analysis (89.24 trials on average for RTs and 96 trials per cell for ERs). ANOVA effect size estimate = $\hat{\eta}_{p}^{2}$; BF_01_ quantifies the evidence for the absence of a main effect or interaction.

**Table S4***Statistical test results of main experiment with gaze cueing trials excluded*

| Effect | *df* | RT | | | | ER | | | |
| --- | --- | --- | --- | --- | --- | --- | --- | --- | --- |
|  |  | *F* | *p* | $\hat{\eta}_{p}^{2}$ | BF_01_ | *F* | *p* | $\hat{\eta}_{p}^{2}$ | BF_01_ |
| Eccentricity | 3, 156 | 34.85 | < .001 | .401 | $3.63\times{10}^{-15}$ | 2.76 | .044 | .050 | 3.76 |
| Face/Gaze Direction | 1, 156 | 42.16 | < .001 | .213 | $2.17\times{10}^{-6}$ | 1.16 | .282 | .007 | 33.01 |
| Motion | 1, 156 | 64.30 | < .001 | .292 | $9.07\times{10}^{-20}$ | 0.08 | .779 | .001 | 25.14 |
| Eccentricity $\times$ Face/Gaze Direction | 3, 156 | 2.43 | .067 | .045 | $2.71\times{10}^{1}$ | 1.39 | .249 | .026 | 54.04 |
| Eccentricity $\times$ Motion | 3, 156 | 4.99 | .002 | .088 | $5.31\times{10}^{-3}$ | 2.46 | .065 | .045 | 11.87 |
| Face/Gaze Direction $\times$ Motion | 1, 156 | 0.17 | .678 | .001 | $1.51\times{10}^{1}$ | 0.25 | .620 | .002 | 17.76 |
| Eccentricity $\times$ Face/Gaze Direction $\times$ Motion | 3, 156 | 2.29 | .081 | .042 | $1.83\times{10}^{1}$ | 1.60 | .192 | .030 | 26.10 |

*Note*. From this set of analyses, we excluded trials in which the target was presented on the left or bottom stimulus to control for gaze cueing effects. ANOVA effect size estimate = $\hat{\eta}_{p}^{2}$; BF_01_ quantifies the evidence for the absence of a main effect or interaction.

**Figure S1***Sample target displays with diagonal gaze cueing*


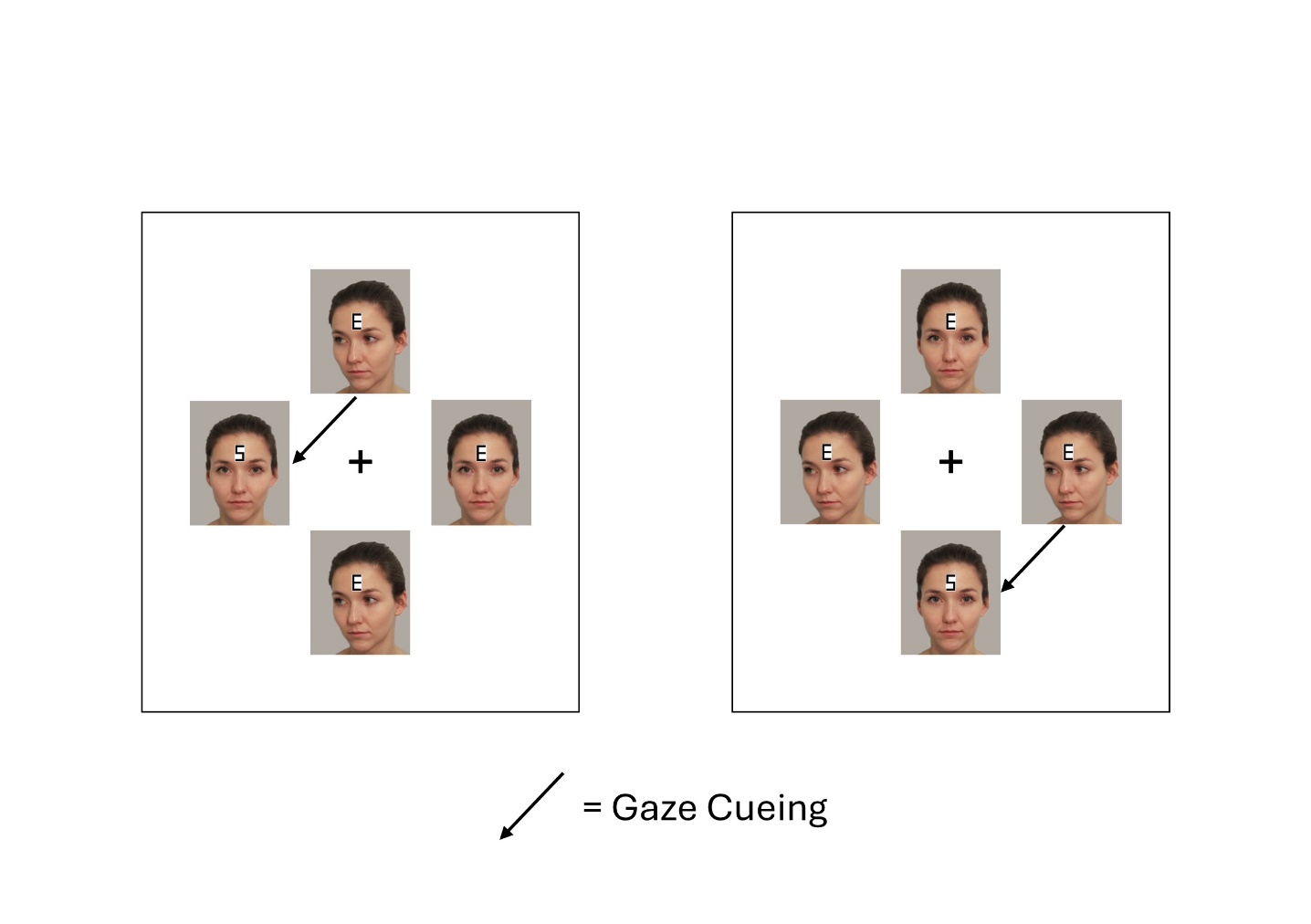


*Note.* Looking slightly to the bottom left, the averted stimulus might direct attention toward the left or bottom position when presented at the top or right position respectively. Such a gaze cueing effect might improve performance for trials with the target stimulus presented at the cued location (as in the examples) beyond the effects of face/gaze direction and sudden-onset motion.

**Figure S2***Results of gaze cueing trials*


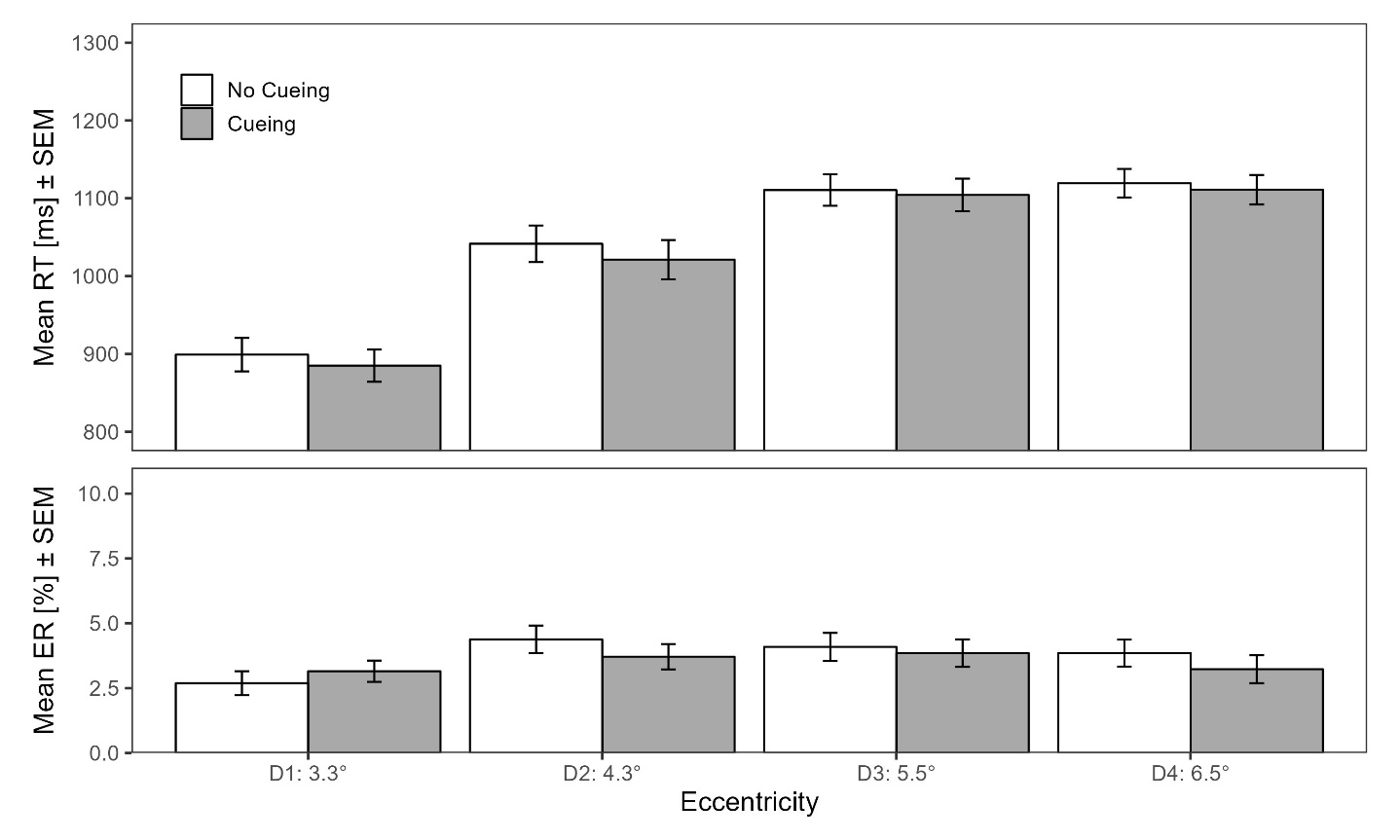


*Note*. Mean RTs and ERs as a function of stimulus presentation eccentricity (x-axis) and diagonal gaze cueing (no cueing vs. cueing). We only considered trials with the target letter on either the left or the bottom stimulus which could be cued by an averted top or right stimulus, respectively. On average, there were 89.24 (*SD* = 5.28) trials per cell for RTs and 96 trials per cell for ERs. Error bars represent standard errors of the mean (SEM).**Figure S3***Results of main experiment with gaze cueing trials excluded*


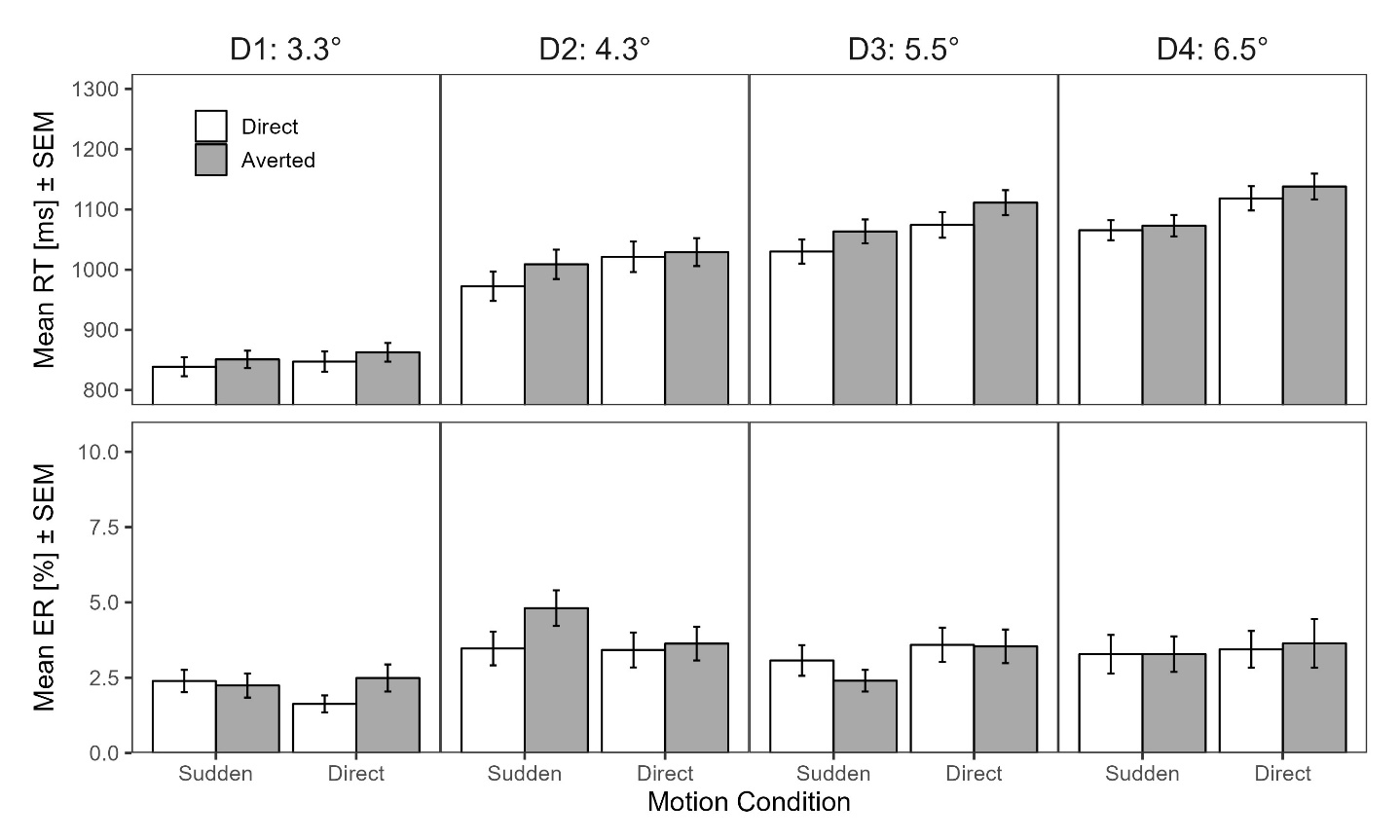


*Note*. Data was prepared in the same way as in the main experiment except that we excluded all trials in which the target letter was presented on the left or bottom stimulus and in which the top or right stimulus was averted, respectively to control for gaze cueing effects within the setup. Mean RT and ER as a function of stimulus eccentricity (in °VA , manipulated across groups, separate panels), motion condition (sudden direction shift vs. static direction, manipulated within-subject, x-axis), and faze/gaze direction (direct vs. averted, manipulated within-subject, white and grey bars, respectively). Error bars represent standard errors of the mean (SEM).
